# A Higher Amount of Nutritional Intake as a Possible Cause of Hyperglycemia in Extremely Premature Infants in Parenteral and Enteral Nutrition at the Tertiary Neonatal Intensive Care Unit

**DOI:** 10.3390/children10101651

**Published:** 2023-10-04

**Authors:** Iza Predanič Drobne, Lilijana Kornhauser Cerar, Vanja Erčulj, Štefan Grosek

**Affiliations:** 1 Neonatology Section, Department of Perinatology, Division of Gynecology and Obstetrics, University Medical Centre Ljubljana, 1000 Ljubljana, Slovenia; predanic.iza@gmail.com (I.P.D.); lilijana.kornhauser-cerar@guest.arnes.si (L.K.C.); 2 Rho Sigma Research & Statistics, 1000 Ljubljana, Slovenia; vanja.erculj@rosigma.si; 3 Faculty of Criminal Justice and Security, University of Maribor, 1000 Ljubljana, Slovenia; 4 Department of Pediatrics, Faculty of Medicine, University of Ljubljana, 1000 Ljubljana, Slovenia

**Keywords:** hyperglycemia, extremely premature infants, nutritional intake, parenteral nutrition, complications of prematurity

## Abstract

Background: This study aimed to find an association between infants who had hyperglycemia and those who did not, those treated with insulin or not and several prenatal and postnatal variables or the suboptimal prescription of parenteral nutrition. Methods: We conducted a retrospective study, which included extremely premature infants (<28 weeks of gestation) admitted to the tertiary NICU, University Medical Centre Ljubljana, between 1 January 2021 and 31 December 2021. Blood glucose measurements, insulin treatment, general characteristics, nutritional data and complications of prematurity were obtained retrospectively from hospital data. RESULTS: There were 21 infants included in the study who did not receive insulin and 17 who were treated with insulin. Infants receiving insulin were younger and lighter compared to the non-insulin treatment group (mean gestational age 178 vs. 188 days; median birth weight 680 g vs. 990 g). The younger insulin group of infants received the same daily number of total macronutrients per kg per day compared to the older non-insulin group, i.e., glucose, lipids and amino acids, as recommended for the gestational age and birth weight. After adjusting for gestational age, no significant association with complications of prematurity was found. Conclusions: The postulated explanation (with the prescription of a higher amount of macronutrients during the first seven days) for hyperglycemia and treatment with insulin in the less mature and lighter infants cannot be supported by the data given.

## 1. Introduction

Hyperglycemia in extremely preterm infants (<28 weeks of gestation) is one of the many metabolic derangements that can occur during treatment in the Neonatal Intensive Care Unit (NICU). The incidence of hyperglycemia ranges between 30% and more than 80% [1,2,3,4,5,6]. It is mainly detected in the first two weeks after birth [2,3,7,8]. Threshold values and the duration of hyperglycemia, when insulin treatment is recommended in extremely premature infants, differ from threshold values in infants and infants later in life. No international threshold of hyperglycemia has yet been accepted for when to start insulin treatment, so hyperglycemia remains a challenge for neonatologists in terms of its definition and treatment. Jagła et al. conducted a study of continuous glucose measurement in interstitial fluid with electrodes and correlated it with glucosuria output. They concluded that the renal glucose threshold is between 8.3 mmol/L and 10.0 mmol/L [9].

The pathogenesis of hyperglycemia in extremely preterm infants is complex, multifactorial and still poorly understood. Factors that mainly contribute to its development are relative hypoinsulinism due to pancreatic beta cell-defective insulin production, peripheral resistance to insulin and hepatocyte dysfunction with persistent gluconeogenesis despite continuous glucose infusion [10,11,12,13,14,15,16]. Some studies have shown that an increased glucose infusion rate and higher lipid intake are independent risk factors for hyperglycemia [2,6,10,11,17,18]. Other risk factors include low gestation age, lower birth weight and the need for inotropes, vasopressors and glucocorticoids [2,4,5,6,7,10,19,20].

Studies have shown an association between early onset hyperglycemia and a higher rate of complications associated with prematurity, i.e., intraventricular hemorrhage (IVH), retinopathy of prematurity (ROP) and necrotizing enterocolitis (NEC), leading to increased morbidity and mortality in extremely preterm infants [4,10,21,22,23,24,25,26,27,28,29,30,31] .The tight control of glucose levels is, therefore, of the utmost importance, as well as starting insulin infusion when the level exceeds a certain threshold [10,19,32].

During the first postnatal days, when enteral feeding is not fully established, continuous parenteral feeding is essential to provide nutrition and prevent hypoglycemia.

The composition of this parenteral mixture is often insufficient to cover the specific needs of the premature infant, which leads to so-called extrauterine growth restrictions (EUGR) and is associated with a greater risk of developing cerebral palsy, behavioral disorders and specific learning difficulties during the school period.

According to the recommendations of the ESPGHAN/ESPEN/ESPR/CSPEN guidelines, premature infants require at least 45–55 kcal/kg/day of energy intake to cover their basic needs in the first few days. After initial weight loss, the goal is to gain 17–20 g/kg per day [33]. Carbohydrates represent the main energy supply in PN. When quantifying glucose levels in parenteral formulations, it is crucial to balance energy needs with the risks of glucose overload. The initial daily intake recommendation is 4 to 8 mg/kg/min. During acute illness, increased catecholamines lowers glucose tolerance, which requires intake adjustments based on glycemic profiles. If, despite restricting glucose intake to 4 mg/kg/min, repeated measures of glucose levels persist above 10–12 mmol/L, then treatment with insulin infusion is initiated [33].

Amino acid intake needs to be initiated within hours of birth to at least 1.5 g/kg/day [33].

It is advisable to begin a continuous infusion of a 20% mixed emulsion 2 days after birth, with a starting infusion rate of at least 2 g/kg/day, which is then quickly raised to a maximum of 4 g/kg/day [33].

Despite the better unification of using nutrients per kg with the computer program, we still found that some infants developed hyperglycemia while some did not. The aim of this study was to evaluate possible associations between the infants who had hyperglycemia and those who did not, those treated with insulin or not and those with prenatal (maternal), postnatal problems (common problems of prematurity) or suboptimal prescription of parenteral nutrition by physicians.

## 2. Materials and Methods

### 2.1. Data Collection

A retrospective analysis was performed in the NICU, Department of Perinatology, Division of Gynecology and Obstetrics, University Medical Centre of Ljubljana. The study was approved by the National Ethics Committee (No: 0120-262/2022/7; date: 10 October 2022). We included all preterm infants (gestational age up to 27 weeks and 6 days) born in the period between 1 January 2021 and 31 December 2021. The exclusion criteria were lethal malformations, chromosomal abnormalities and death within the first week after birth. Clinical data were extracted from our database regarding prematurity complications (intraventricular hemorrhage (IVH), cystic periventricular leukomalacia (cPVL), bronchopulmonary dysplasia (BPD), necrotizing enterocolitis (NEC), severe retinopathy of those in prematurity who needed ophtalmological interventions (ROP), sepsis, systemic inflammatory response syndrome (SIRS) and pneumothorax), nutritional intake and daily glucose measurements. We included data on maternal risk factors (maternal age, gravidity, gestational hypertension, (pre)eclampsia, gestational diabetes mellitus, chorioamnionitis and abruption of the placenta) and data regarding labor and measures taken in the delivery room.

### 2.2. Hyperglycemia—Definition, Measurements and Threshold

All available retrospective glucose measurements were obtained from our database. Glucose measurements were performed every 6 h in the first few days. If no hyperglycemia was detected, the measurement interval was extended to one measurement daily or as required if measurements of another electrolyte or blood gas analysis were needed. The glucose values were analyzed out of plasma samples collected from different origins (venous blood, taken from the peripheral vein or venous umbilical catheter, arterial blood, taken from the arterial umbilical catheter or occasionally from a capillary sample) by a blood gas analyzer. We defined hyperglycemia as a one-time measurement above 8 mmol/L or if, at the time of sample acquisition, the infant was already receiving an insulin infusion due to hyperglycemia. When hyperglycemia was detected, the continuous glucose infusion rate was reduced, and the glucose concentration was carefully monitored. If the plasma concentration remained highly elevated (10–12 mmol/L or higher), insulin infusion was started. During the study period, there were no national guidelines for insulin treatment, so insulin treatment was given based on clinical judgment.

### 2.3. Nutrition—Enteral, Parenteral, Total

Parenteral nutrition (PN) was introduced to all infants immediately after birth and acquired adequate vessel access. Within 24 h after birth, we gradually started enteral nutrition, which was given intermittently every three hours via a nasogastric tube. The required PN intake was calculated with the help of our computer software, which calculated the required fluid, macronutrient (carbohydrates, lipid, amino acids) and micronutrient (electrolytes, vitamins) intake in extremely premature infants whilst taking into account enteral nutrition [33]. The neonatologist is presented with a simple web interface written in HTML and JavaScript. Basic data (ID number, name, date of birth, gestational age and weight) entry is required for calculation. The program calculates and displays specific nutrient intake based on Tsang et al.’s 2005 guidelines in terms of age in days, gestational age and birth weight [33]. After calculation, the desired volume and amount of each nutrient are filled in by the neonatologist. Any deviations from the recommended values are automatically detected, thus eliminating most of the human errors involved in manually calculating the parenteral nutrition composition. Before the final composition of PN is determined, the amount of enteral nutrition and any arterial catheter infusion are added to the equation. The program also considers the stability of combined nutrients in the PN solution and alerts the neonatologist of any disproportion in nutrients. The data are then sent to the back-end script written in Python, which submits an online order to the pharmacy. The preparation of PN is conducted centrally by our pharmacist at the Department of Pharmacy with the use of a computerized system and under strict aseptic control. The data are stored in a database, making it available for later use and further statistical analysis. The web interface setup is beneficial since no installation on the client side is necessary, and only a web browser is required.

We analyzed the amount and composition of parenteral and enteral nutrition received for each child up to and including the 7th day of their stay in the NICU. Afterward, we calculated the average daily intake of macronutrients for each individual (g/kg/day) and the sum of the volume of enteral nutrition in the first week (mL/kg/week).

### 2.4. Statistical Analysis

We compared two subgroups: (1) extremely premature infants who did not require insulin infusion up to 7 days after birth and (2) extremely premature infants who required insulin infusion for the treatment of hyperglycemia up to 7 days after birth.

Categorical variables were described with frequencies and percentages, and continuous variables with means and standard deviations or, in the case of non-normality, with medians and interquartile ranges. The normality of distribution was tested using the Shapiro–Wilk test. To test the association between maternal pregnancy-related characteristics, the infant’s birth characteristics, nutritional variables, complications of prematurity, insulin application and univariate logistic regression were used.

For nutritional variables and complications, adjusted odds ratios were calculated with gestational age included as a control variable (the two cohort groups differed in gestational age). In the case of zero units within the contingency table, a likelihood ratio test was used to test the association between variables. Associations with *p* < 0.05 were treated as statistically significant.

## 3. Results

During the study period, 50 extremely preterm newborn infants (below 28 weeks of gestation) were admitted to the ICU. Of these fifty infants, six died and six were lost for inclusion and were not included in the study. Finally, 38 extremely premature infants were included in the study. Twenty-one (55.3%) infants included in the study did not receive insulin, and seventeen (44.7%) had hyperglycemia and were treated with insulin. The mothers of the two cohorts of infants did not differ significantly in statistics on pregnancy-related complications and diseases (Table 1). The infants without insulin treatment were statistically significant (*p* = 0.002) older—the mean (SD) gestational age was 188 (5) days—in comparison to younger infants receiving insulin—the mean (SD) gestational age was 178 (8) days. The median (IQR) birth weight of infants not receiving insulin was 990 (820–1050) g, while half of the infants receiving insulin weighed 680 g or less (IQR: 620–740). This difference was statistically significant (*p* = 0.005).

The two cohorts of infants differed statistically and significantly in the mean daily volume of received enteral nutrition or in the first week when not controlling for gestational age but ceased to differ when controlling for gestational age (Table 2). The infants treated with insulin received a lower mean daily volume of enteral nutrition in the first week in comparison to infants not treated with insulin, but the difference was due to the difference in gestational age.

As has been said, the infants receiving insulin were younger (lower gestational age) and lighter (lower birth weight) compared to the non-insulin treatment group (mean gestational age 178 vs. 188 days; median birth weight 680 g vs. 990 g). The younger, insulin (lower gestational age, lower birth weight) group of infants received the same daily number of total macronutrients per kg per day compared to the older, non-insulin group (higher gestational age, higher birth weight), i.e., glucose (*p* = 0.457), lipids (*p* = 0.461) or amino acids (*p* = 0.184), as recommended for gestational age and birth weight. When controlling for gestational age, which is related to birth weight, data showed no differences.

The same applied to the received daily amount of nutrients in the form of glucose, lipids and amino acids received from parental nutrition.

The higher share of infants receiving insulin had ROP (13; 76.5%) in comparison to the group not treated with insulin (4; 19%), but this difference was significant before controlling for the gestational age of infants (*p* = 0.001), while, after adjusting for gestational age, this difference was not statistically significant (*p* = 0.270). The median (IQR) length of hospitalization was longer for infants in the insulin-receiving group (80 (66–89) days) than for infants not receiving insulin (58 (51–73) days). This difference was statistically significant before adjusting for gestational age (*p* = 0.031) and not after adjusting for the gestational age of infants (*p* = 0.973). No statistically significant association between other after-birth complications and the cohort group was found (Table 3).

## 4. Discussion

This retrospective single-center study, which included 38 extremely preterm infants (<28 weeks of gestation), shows that severe hyperglycemia is common in the first week after birth and that it is associated with lower gestational age and birth weight. However, we were unable to confirm that an inappropriate calculation of daily macronutrient requirements, despite the use of a computer program for the daily calculations of allowances of macronutrients, could be the cause of hyperglycemia. There were 17 (44.7%) infants that were treated with insulin to maintain normoglycemia. These data correlate with other studies. Bermick et al. reported that 35% of 216 extremely low birth weight infants (<1000 g) had a glucose concentration of more than 11.0 mmol/L in the first 10 postnatal days, requiring insulin treatment [31].

Maternal and child characteristics with clinical outcomes are shown in Table 1 and Table 3. Infants receiving insulin were statistically and significantly younger (lower gestational age) and lighter (lower birth weight) (mean gestational age 178 vs. 188 days; median birth weight 680 g vs. 990 g) and required mechanical ventilation more often (76.5% vs. 28.6% of infants) compared to those who did not need insulin treatment. No difference in shares of small for gestational age was observed (OR 4.2; 95% CI 0.88–19.94; *p* = 0.071).

Similar findings have been observed in former studies conducted by Beardsall et al. and Stensvold et al. [2,18] regarding gestational age, birth weight and the need for mechanical ventilation. Their findings included the higher use of vasopressors and inotropes in groups with severe hyperglycemia.

Infants treated with insulin received a lower mean daily volume of enteral nutrition in the first week in comparison to infants not treated with insulin, but this difference was due to the difference in gestational age. The two groups of infants, including both insulin and non-insulin groups, did not differ in the daily amount of total nutrients they received in enteral and parenteral nutrition, glucose, lipids and amino acids, after controlling for gestational age, which meant that we prescribed too many macronutrients to the younger group of infants. According to guidelines, our computer software is programmed to calculate fewer macronutrients for younger and lighter extreme premature infants born at 25 weeks than infants born up to 28 weeks of gestational age. However, it includes and allows the lower minimum and upper maximum limits of nutrients that can be prescribed per body weight and gestational age and for each day after birth separately.

As has been said, infants receiving insulin were younger and lighter than the non-insulin treatment group (mean gestational age 178 vs. 188 days; median birth weight 680 g vs. 990 g). The younger group of infants received higher daily limits of total nutrients, as recommended in enteral and parenteral nutrition, compared to the older group, i.e., glucose (*p* = 0.457), lipids (*p* = 0.461) or amino acids (*p* = 0.184), even when controlling for gestational age.

These results are supported by the earlier findings of Stensvold et al., who described a higher proportion of hyperglycemia among infants who received early enhanced parenteral nutrition [17]. In this study, which included 580 extremely preterm infants born at <27 weeks of gestational age, they concluded that a strict glucose infusion strategy was associated with a significant reduction in the prevalence of early hyperglycemia [18]. Chacko et al. conducted a small study on eight infants below 30 weeks of gestational age and reported a positive correlation between the glucose infusion rate and plasma glucose concentration. [11] On the other hand, Beardsall et al. and Zamir et al., in their studies, did not find any significant correlation between glucose infusion rates and plasma glucose concentration [2,6].

Some previous studies have already shown a correlation between hyperglycemia and mortality, IVH, BPD, NEC, ROP and other complications with prematurity [4,23,24,25,26,27,28,29,30,31]. We found that ROP was more frequent in the group that received insulin treatment, but this difference was not statistically significant after adjustments were made for gestational age (aOR 3.03; 95% CI 0.42–21.78; *p* = 0.27). No correlation between other complications of prematurity and the need for insulin treatment was found.

The benefits of our study included the use of computer software designed for the calculation of parenteral nutrition, which enabled the controlled infusion of nutrition. However, because calculations of daily allowances permit a variation between the lower and upper limits of daily allowances of macronutrients, many physicians, in striving for the better growth of infants, have probably chosen the upper limits of daily allowances for macronutrients. Stensvold et al. already described a higher proportion of hyperglycemia among infants who received early enhanced parenteral nutrition in their study [17].

The limitations of our study are the small number of included infants. Despite the long history and knowledge of prescribing total parenteral nutrition for extremely premature infants, we lack clear national guidelines on when to start insulin treatment and rely only on various international guidelines, which vary in their recommendations.

## 5. Conclusions

The postulated explanation (with a prescription of a higher amount of macronutrients during the first seven days) for hyperglycemia and treatment with insulin in the less mature and lighter infants cannot be supported by the data given. Further studies in larger groups of infants together with a control group are needed to find more conclusive data on this matter.

## Figures and Tables

**Table 1:** Association between maternal and child risk factors and insulin application (results of univariate logistic regression).

	No Insulin Application Group (*n* = 21)	Insulin Application Group (*n* = 17)	OR (95% CI)	*p*
**Maternal characteristics**				
Gestational diabetes mellitus	5 (23.8)	2 (11.8)	0.43 (0.07–2.54)	0.350
Preeclampsia	2 (9.5)	4 (23.5)	2.92 (0.47–18.37)	0.253
Hypertension	1 (4.8)	1 (5.9)	1.25 (0.07–21.58)	0.878
Chorioamnionitis	7 (33.3)	5 (29.4)	0.83 (0.21–3.32)	0.796
**Child characteristics**				
Female gender	8 (38.1)	11 (64.7)	2.98 (0.79–11.25)	0.107
Gestational age (days; mean ± SD)	188 ± 5	178 ± 8	0.78 (0.67–0.91)	0.002
Birth weight (g; median (IQR))	990 (820–1050)	680 (620–740)	0.98 (0.97–1)	0.005
SGA	3 (14.3)	7 (41.2)	4.2 (0.88–19.94)	0.071
Prenatal application of glucocorticoids	19 (90.5)	14 (82.4)	0.49 (0.07–3.34)	0.468
Prenatal application of antibiotics	8 (38.1)	6 (35.3)	0.89 (0.23–3.35)	0.859
Resuscitation in delivery room				
No	5 (23.8)	3 (17.6)	1	
Hand bagging	13 (61.9)	12 (70.6)	1.54 (0.3–7.87)	0.605
Intubation	3 (14.3)	2 (11.8)	1.11 (0.11–10.99)	0.928
Surfactant	14 (66.7.	15 (88.2)	3.75 (0.66–21.2)	0.135
Glucocorticoids	6 (28.6)	7 (41.2)	1.75 (0.45–6.77)	0.417
Vasopressors	1 (4.8)	2 (11.8)	2.67 (0.22–32.23)	0.44
Respiratory support				0.009 ^a^
No	1 (4.8)	0 (0)		
Noninvasive	14 (66.7)	4 (23.5)		
Invasive	6 (28.6)	13 (76.5)		

a = likelihood ratio test; SGA—small gestational age.

**Table 2:** Association between nutrition and insulin application in the first week (results of univariate logistic regression and adjusted logistic regression model for gestational age; aOR = adjusted odds ratio).

	No Insulin Application Group (*n* = 21)	Insulin Application Group (*n* = 17)	OR (95% CI)	*p*	aOR (95% CI)	*p*
**Mean daily volume of enteral nutrition**						
Mean ml/kg/day (median (IQR))	22.8 (6–30.2)	11.7 (5.6–15.1)	0.93 (0.87–1)	0.039	0.97 (0.9–1.05)	0.414
**Sum of volume of enteral nutrition in the first week** ml/kg/week (median (IQR))	182 (48–242)	94 (45–121)	0.99 (0.98–1)	0.039	1 (0.99–1.01)	0.414
**Mean total amount of nutrients in enteral and parenteral nutrition in the first week ** Mean glucose g/kg/day (mean ± SD)	7.9 ± 0.9	7.7 ± 0.9	0.76 (0.37–1.57)	0.457	1.67 (0.59–4.68)	0.331
Mean amino acids g/kg/day (median (IQR))	3.3 (3.1–3.6)	3.5 (3.3–3.8)	3.88 (0.52–28.69)	0.184	5.27 (0.28–100.25)	0.269
Mean lipids g/kg/day (mean ± SD)	2.6 ± 0.4	2.5 ± 0.4	0.52 (0.–2.95)	0.461	1.09 (0.1–11.83)	0.941
Mean Kcal/g N2 (mean ± SD)	74.9 ± 11.9	74.3 ± 8	0.99 (0.93–1.06)	0.862	1.02 (0.93–1.11)	0.704
**Mean amount of nutrients in parenteral nutrition in the first week**						
Mean glucose g/kg/day (mean ± SD)	6.1± 1.2	6.4± 1.1	1.25 (0.69–2.26)	0.453	1.32 (0.58–2.98)	0.511
Mean amino acids g/kg/day (mean ± SD)	2.5± 0.4	2.7 ± 0.4	3.32 (0.66–16.74)	0.146	1.37 (0.15–12.36)	0.778
Mean lipids g/kg/day (mean ± SD)	1.6 ± 0. 4	1.7± 0.3	3.35 (0.55–20.46)	0.19	1.34 (0.13–14.28)	0.81

IQR—interquartile range; SD—standard deviation.

**Table 3:** Association between complications, length of stay in the NICU and insulin application (results of univariate logistic regression and adjusted logistic regression model for gestational age; aOR = adjusted odds ratio).

	No Insulin Application Group (*n* = 21)	Insulin Application Group (*n* = 17)	OR (95% CI)	*p*	aOR (95% CI)	*p*
Pneumothoracs	1 (4.8)	1 (5.9)	1.25 (0.07–21.58)	0.878	1.34 (0.01–231.67)	0.912
NEC	1 (4.8)	1 (5.9)	1.25 (0.07–21.58)	0.878	0.09 (0–2.95)	0.177
Late sepsis	2 (9.5)	6 (35.3)	5.18 (0.89–30.25)	0.068	2.23 (0.21–24.06)	0.509
SIRS	4 (19)	5 (29.4)	1.77 (0.39–8)	0.458	1.43 (0.24–8.6)	0.695
ROP	4 (19)	13 (76.5)	13.81 (2.89–65.91)	0.001	3.03 (0.42–21.78)	0.270
BPD	17 (81.0)	16 (94.1)	3.76 (0.38–37.37)	0.258	1.33 (0.08–23.22)	0.844
ICH	5 (23.8)	6 (35.3)	1.75 (0.42–7.17)	0.44	1.4 (0.22–8.76)	0.717
PVL	2 (9.5)	0 (0)		0.117 ^a^		
No. of days in NICU (median (IQR))	58 (51–73)	80 (66–89)	1.05 (1–1.09)	0.031	1 (0.95–1.06)	0.973

a = likelihood ratio test. NEC—necrotizing enterocolitis; SIRS—systemic inflammatory response syndrome; ROP—retinopathy of prematurity; BPD—bronchopulmonary dysplasia; ICH—intracranial hemorrhage; PVL—periventricular leukomalacia; NICU—neonatal intensive care unit; IQR—interquartile ratio.

## Data Availability

Data are not available due to privacy or ethical restrictions.
